# Heritability of the limbic networks

**DOI:** 10.1093/scan/nsv156

**Published:** 2015-12-28

**Authors:** Sanja Budisavljevic, Jamie M. Kawadler, Flavio Dell'Acqua, Frühling V. Rijsdijk, Fergus Kane, Marco Picchioni, Philip McGuire, Timothea Toulopoulou, Anna Georgiades, Sridevi Kalidindi, Eugenia Kravariti, Robin M. Murray, Declan G. Murphy, Michael C. Craig, Marco Catani

**Affiliations:** ^1^Department of Forensic and Neurodevelopmental Sciences, and; ^2^Natbrainlab, Centre for Neuroimaging Sciences, Institute of Psychiatry, Psychology and Neuroscience, King’s College London, London SE5 8AF, UK,; ^3^NEMo Laboratory, Department of General Psychology, University of Padova, 35131 Padova, Italy,; ^4^Social, Genetic and Developmental Psychiatry Research Centre,; ^5^Department of Psychological Medicine, and; ^6^Department of Psychosis Studies, Institute of Psychiatry, Psychology and Neuroscience, King’s College London, London SE5 8AF, UK,; ^7^Department of Psychology, and; ^8^State Key Laboratory of Brain and Cognitive Sciences, The University of Hong Kong, Hong Kong, Hong Kong, and; ^9^National Autism Unit, South London and Maudsley NHS Foundation Trust, Beckenham, UK.

**Keywords:** heritability, limbic system, cingulum, uncinate fasciculus, fornix, diffusion tractography

## Abstract

Individual differences in cognitive ability and social behaviour are influenced by the variability in the structure and function of the limbic system. A strong heritability of the limbic cortex has been previously reported, but little is known about how genetic factors influence specific limbic networks. We used diffusion tensor imaging tractography to investigate heritability of different limbic tracts in 52 monozygotic and 34 dizygotic healthy adult twins. We explored the connections that contribute to the activity of three distinct functional limbic networks, namely the dorsal cingulum (‘medial default-mode network’), the ventral cingulum and the fornix (‘hippocampal-diencephalic-retrosplenial network’) and the uncinate fasciculus (‘temporo-amygdala-orbitofrontal network’). Genetic and environmental variances were mapped for multiple tract-specific measures that reflect different aspects of the underlying anatomy. We report the highest heritability for the uncinate fasciculus, a tract that underpins emotion processing, semantic cognition, and social behaviour. High to moderate genetic and shared environmental effects were found for pathways important for social behaviour and memory, for example, fornix, dorsal and ventral cingulum. These findings indicate that within the limbic system inheritance of specific traits may rely on the anatomy of distinct networks and is higher for fronto-temporal pathways dedicated to complex social behaviour and emotional processing.

## Introduction

Socio-emotional functioning and behaviour depend on the coordinated activity of the limbic system, composed of specific cortical and subcortical regions interconnected by white matter tracts (Catani *et al*., 2012). These tracts are implicated in a variety of neuropsychiatric disorders when deficits in emotion regulation, social behaviour and memory are present (Kubicki* et al.*, 2003; Mori* et al.*, 2007; Metzler-Baddeley* et al.*, 2011, 2012; Nortje* et al.*, 2013; Douet and Chang, 2014; Ameis and Catani, 2015; Whitford* et al.*, 2015).

Recently, our group has proposed a revised model of the limbic system, with limbic tracts connecting three distinct, yet partially overlapping functional networks (Catani* et al.*, 2013a). The first functional network, the ‘dorsomedial default-mode network’, consists of a group of medial regions interconnected by dorsal cingulum, and is involved in mental activities associated with the ‘resting state’ such as introspection, social, moral and affective processing. The dorsal cingulum is the uppermost component of the cingulum bundle, and runs along the length of the cingulate gyrus. Altered activations of the default-mode network are observed in various neuropsychiatric disorders (Broyd* et al.*, 2009), including schizophrenia (Koch *et al.*, 2013), psychopathy (Sethi *et al.*, 2015), depression (Sheline *et al.*, 2009), and so on. The second network, the ‘hippocampal-diencephalic-retrosplenial’ network, encompasses the fornix and ventral cingulum, which are dedicated to memory and spatial orientation. The fornix is a small projection tract with a commissural component, which links hippocampus with the mammilary body, the anterior thalamic nuclei and the hypothalamus (Aggleton, 2008). The ventral cingulum is the posterior ventral component of the cingulum bundle running within the parahippocampal gyrus and retrosplenial cingulate gyrus (Catani* et al.*, 2013a). Lesions of this network are associated with memory (Valenstein* et al.*, 1987) and spatial orientation deficits (Vann* et al.*, 2009) and are implicated in neurodegenerative disorders, such as Alzheimer’s disease (Buckner* et al.*, 2005), temporal lobe epilepsy (Concha* et al.*, 2010), mild cognitive impairment (Nestor* et al.*, 2003), and schizophrenia (Fitzsimmons* et al.*, 2014). The third functional network, the ‘temporo-amygdala-orbitofrontal network’, is involved in the integration of visceral and emotional states with cognition and behaviour (Mesulam, 2000). Its main tract is the uncinate fasciculus that runs from the anterior part of the temporal lobe, parahippocampal gyrus, uncus and amygdala to the orbital and polar frontal cortex (Catani* et al.*, 2002). The uncinate fasciculus has been linked to different aspects of social development (Elison* et al.*, 2013), recognition of complex emotions (Fujie* et al.*, 2008), semantic cognition (Catani *et al.*, 2013a,b; Catani and Mesulam, 2008; Catani and Bambini, 2014), and social behavior (D’Anna *et al.*, in press). Damage to this network is related to deficits in socio-emotional processing in disorders such as autism (Pugliese* et al.*, 2009; Kumar* et al.*, 2010; Thomas* et al.*, 2011; Catani *et al.*, in press), psychopathy (Craig* et al.*, 2009), borderline personality disorder (Lischke *et al.*, 2015), apathy (Hollocks *et al.*, 2015), mood disorders (McIntosh* et al.*, 2008; Carballedo* et al.*, 2012; Zhang *et al.*, 2012), obsessive-compulsive disorder (Peng* et al.*, 2014), anorexia nervosa (Lipsman *et al.*, 2015), and alcoholism (Schulte *et al.*, 2012). Overall, individual differences in socio-emotional behaviour and vulnerability to specific neuropsychiatric disorders have been associated with the variability in the structure of the three limbic networks listed above.

Hence, understanding the relative influence of genetic and environmental factors on the structure of limbic tracts can help to clarify the biological mechanisms underlying predisposition to vulnerability and resilience to neuropsychiatric disorders. The classical twin design provides a unique method for establishing heritability by comparing monozygotic (MZ) and dizygotic (DZ) twins raised in the same family. The heritability is reflected in the difference in correlations between MZ and DZ twins, because MZ twins share all their genetic sequence while DZ twins share approximately 50% of their genes. If MZ twins resemble each other more than DZ twins, the trait can be assumed to be heritable. Furthermore, using structural equation modelling (SEM) the phenotypic variance can be parsed into genetic and environmental components (shared environmental and specific environmental factors, which include the measurement error) (Plomin* et al.*, 2013).

Using twin design, limbic system functions such as social cognition (Scourfield *et al.*, 1999), associative memory (Thapar* et al.*, 1994), aggressive behaviour (Craig and Halton, 2009), prosocial behaviour and empathy (Matthews* et al.*, 1981; Rushton* et al.*, 1984, 1986; Knafo and Israel, 2009), antisocial behaviour (Viding *et al.*, 2005), parenting (Losoya *et al.*, 1997), anxiety-related personality characteristics (Stein *et al.*, 2002) and personality measures such as psychoticism, extraversion, and neuroticism (Plomin *et al.*, 1994; Eaves *et al.*, 1999) are found to be moderately to highly heritable throughout lifespan. However, certain traits are more heritable than others, for example, the personality traits (46-51%) and spatial reasoning (40%) compared to memory (22%) (Plomin *et al.*, 1994). Moreover, heritability seems to be age dependent, and the twin literature on prosocial traits, empathy, externalizing behaviours, but also anxiety and depressive symptoms, indicates the increasing importance of genes with increasing age, while shared environmental effects decrease in importance from early childhood to adulthood (Bergen *et al.*, 2007; Ebstein* et al.*, 2010).

Structural brain imaging studies in healthy twins report moderate to high heritability for limbic grey matter regions such as posterior cingulate gyrus (51-75%), hippocampus (64–71%), and amygdala (55–71%) (Wright* et al.*, 2002; Hulshoff Pol* et al.*, 2006; Kremen* et al.*, 2010). Hence, it is not surprising that limbic structures act as putative endophenotypes in different disorders such as schizophrenia (Weinberger* et al.*, 1992; McDonald *et al.*, 2004; Rijsdijk* et al.*, 2005; van Haren* et al.*, 2012; Roalf* et al.*, 2015), bipolar disorder (McDonald *et al.*, 2004), epilepsy (Scanlon* et al.*, 2013; Alhusaini* et al.*, 2015), and dementia (Järvenpää* et al.*, 2004).

Little is known about genetic and environmental influences on specific limbic white matter tracts. Advances in diffusion tensor imaging (DTI) tractography permit to reliably reconstruct and study specific white matter pathways *in vivo*. To date, only three studies have used DTI tractography to explore genetics of limbic pathways in childhood. One study on neonates (Lee* et al.*, 2015) reported high heritability of the anatomy of fornix and ventral cingulum (hippocampal-diencephalic-retrosplenial network), compared to the uncinate fasciculus and dorsal cingulum (temporo-amygdala-orbitofrontal and medial default-mode networks) that are known to mature later in life (Lebel* et al.*, 2008). With increasing age from early to late childhood, a genetic effect seems to significantly decrease for the fornix, while it moderately increases for the uncinate and more strongly for the cingulum (Brouwer* et al.*, 2010, 2012). Previous DTI studies on adults have been conducted using voxel-based methods that lack the ability to answer questions regarding specific fibre tracts. Nevertheless, these studies suggest low heritability for the microstructure of fornix (Jahanshad* et al.*, 2013), moderate to high for cingulum (Kochunov* et al.*, 2010; Gatt* et al.*, 2012; Jahanshad* et al.*, 2013; Shen* et al.*, 2014), and high for uncinate fasciculus (Gatt* et al.*, 2012). Overall, studies indicate that heritability of limbic pathways is heterogeneous and changes with increasing age (Lenroot* et al.*, 2009).

To the best of our knowledge, there is no DTI study that has explored heritability of limbic tracts in healthy adults using tractography. Here we used DTI tractography and univariate twin modelling to study the heritability of specific limbic pathways in healthy adults. We compared the dorsal cingulum (medial default-mode network), the ventral cingulum and the fornix (hippocampal-diencephalic-retrosplenial network) and the uncinate fasciculus (temporo-amygdala-orbitofrontal network) in 52 MZ and 34 DZ adult twins. The contributions of genes and environment were studied on multiple tract-specific measures including volume, fractional anisotropy and mean diffusivity that reflect different aspects of the underlying anatomy, microarchitecture, and biology.

## Methods

### Participants

In this study, we included 86 healthy right-handed adult twins, 26 MZ and 17 DZ twin pairs, recruited from a volunteer twin register at the Institute of Psychiatry, Psychology and Neuroscience (London, UK) and by national media advertisements. Mean age for MZ twins was 35.5 years (age range 21–56 years), and 42.5 years (age range 20–62 years) for DZ twins. All DZ twin pairs included in this study were same-sex pairs. Opposite-sex pairs were excluded to avoid inflation of heritability estimates due to an overall lower DZ correlation due to qualitative sex differences (Stromswold, 2001). Of the 26 MZ twin pairs, 9 pairs were male and 17 female. Of the 17 DZ twin pairs, 4 pairs were male and 13 were female. Other exclusion criteria included a history of neurological disorder or systemic illness with known neurological complications, significant head injury associated with loss of consciousness for more than one minute and current harmful substance use or dependence (defined as within the last 12 months). A UK Multicentre Research Ethics Committee approved the study, and all the participants gave written informed consent before participating. Due to movement and scanner artefacts we excluded two DZ twin pairs from the cingulum analysis, four DZ twin pairs from the uncinate fasciculus analysis, and two MZ twin pairs and one DZ twin pair from the fornix analysis.

### Data acquisition and tractography analysis

DTI data were acquired using a GE Signa 1.5-T LX MRI system (General Electric, Milwaukee, WI) with maximum gradient amplitude of 40 mT/m and an acquisition sequence fully optimized for DTI and tractography of white matter. All images were acquired from the whole brain, with sections parallel to the anterior–posterior commissure line. Using a peripherally gated echo planar imaging pulse sequence, each DTI-MRI volume was acquired from 60 contiguous 2.5 mm thick slices with a field of view of 240 × 240 mm and an acquisition matrix size of 96 × 96 pixels, giving an isotropic voxel size of 2.5 mm × 2.5 mm × 2.5 mm. Echo time was 102 ms. Acquisitions were peripherally gated with an effective repetition time of 15 R–R intervals. At each location, 7 images were acquired without diffusion weighting, together with 64 images with a weighting of 1300 s/mm^2^ applied along directions uniformly distributed in space.

Diffusion data were analysed using ExploreDTI (Leemans and Jones, 2009). Following correction for eddy current distortions and subject motion, the diffusion tensor (DT) was estimated using a non-linear least square methods and fractional anisotropy (FA) and mean diffusivity (MD) maps were estimated. Whole brain tractography was performed using a Euler integration to propagate streamlines following the directions of the principal eigenvector with a step size of 0.5 mm. Tractography was started in all brain voxels with FA > 0.2. Streamlines were tracked until the FA of the tensor was above an FA threshold of 0.2 or the curvature (i.e. the angle between two consecutive steps) was less than 30°.

### Virtual dissections of the limbic system pathways

The tractography data were visualized using TrackVis (Massachusetts General Hospital, Boston, MA) to perform the virtual dissection of white matter bundles.

#### Uncinate fasciculus

We used a two-regions of interest (ROI) approach to dissect the uncinate fasciculus, in line with the method previously described by our laboratory (Catani and Mesulam, 2008). The first ROI was placed in the white matter of the anterior floor of the external/extreme capsule on contiguous axial slices, while the second ROI was placed in the white matter of the anterior temporal lobe.

#### Fornix

We used a one-ROI approach to dissect the fornix, as described previously by our laboratory (Catani and Thiebaut de Schotten 2008). This choice was made because the fornix contains a commissural component known as the hippocampal commissure, and thus we did not separate the left and the right columns. Thus, a single ROI is placed around the body of the fornix on contiguous coronal slices inferior to the corpus callosum. To better visualize the entire course of the fornix into the temporal lobe, additional regions around the fimbriae of the left and right side were included in the ROI.

#### Dorsal and ventral cingulum

**Fig. 1.**
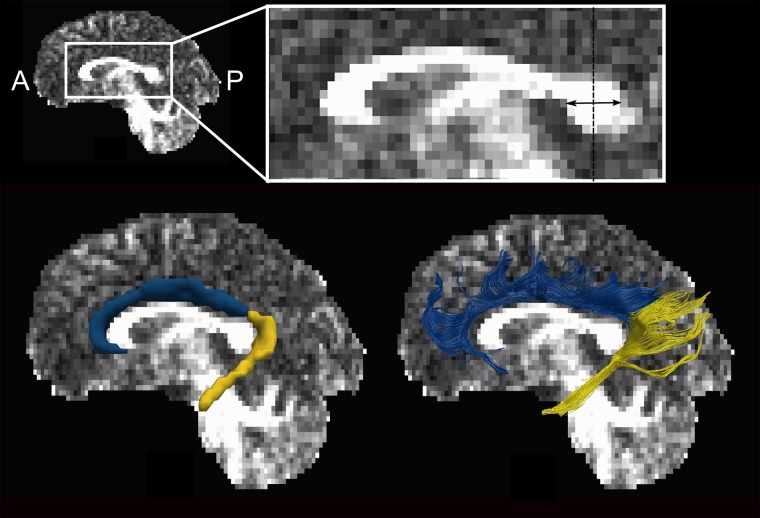
Dissected dorsal (blue) and ventral (yellow) components of the cingulum bundle. The vertical midline of the splenium of the corpus callosum separates the uppermost anterior coronal slices of the dorsal cingulum from the lowermost slices of the ventral cingulum. When the width of the splenium is an even number of voxels and cannot be halved easily, the consistent method is to select the first voxel of the posterior half of the splenium. This slice is included in the dorsal cingulum ROI (blue).

In this study, we introduce a novel method for segmenting the cingulum bundle into its dorsal and ventral components, in contrast to the one-ROI approach for dissecting the whole cingulum bundle described previously by our group (Catani and Thiebaut de Schotten 2008). Previous studies have attempted segmentation based on a single ROI approach (Sun *et al.*, 2003; Wu *et al.*, 2010), a two-ROI approach investigating either the long fibres of the dorsal cingulum or ventral cingulum (Zhang *et al.*, 2007; Nakata *et al.*, 2009; Takei *et al.*, 2009; Kamagata *et al.*, 2012), or dissection of the cingulum based on multiple ROIs placed at specific points along the fasciculus (Catani *et al.*, 2002; Kim *et al.*, 2006; Catheline *et al.*, 2010). Importantly, the cingulum bundle consists of fibres of different length including the long fibres and the short U-shaped fibres. For that reason, we used an updated one-ROI approach from Catani and Thiebaut de Schotten (2008), which identifies both types of cingulum fibers, unlike other two-ROI approaches that exclude the majority of the short U-shaped fibres from the analysis. Thus, we used a one-ROI approach separately for both dorsal and ventral cingulum as following. First, a vertical midline bisecting the splenium of the corpus callosum was delineated on a midsagittal coronal slice. For the dissection of the dorsal cingulum, a single ROI was placed on contiguous coronal slices around the cingulum bundle anterior to the vertical midline of the splenium and superior to the body of the corpus callosum. For the ventral cingulum, a single ROI was delineated on contiguous slices posterior to the vertical midline of the splenium and inferiorly along the medial temporal lobe/parahippocampal gyrus (Figure 1). Thus, besides distinguishing dorsal and ventral cingulum, our novel approach allowed for visualization not only of the long association fibres, but also of the U-shaped local fibres that connect the neighbouring gyri and represent the majority of the cingulum fibres.

### Statistical analysis

For each limbic tract, namely fornix and bilateral dorsal cingulum, ventral cingulum, and uncinate fasciculus, the number of voxels touched by the tract streamlines was calculated as a surrogate measure of the tract volume (referred from now on as Volume), together with average tract-specific measures of FA and MD. We confirmed that these quantitative measures were normally distributed, and that there was homogeneity of means and variances across the measures (using paired *t*-tests and Levene’s test, respectively).

A comparison of intra-class correlation coefficients (ICCs) of DTI measures in MZ and DZ twins provided initial descriptive statistics on the presence of genetic effects. An MZ ICC that is at least twice as large as a DZ ICC indicates that this measure is largely influenced by genetic factors. An MZ correlation larger than, but less than twice the DZ correlation, indicates the significant effect of shared environmental factors (all non-genetic factors that make family members more alike).

Prior to quantitative genetic model fitting of the extracted diffusion measures from the limbic connections, age, sex and handedness were regressed out using SPSS, and residuals standardized for subsequent analysis. On that note, we have recently reported the absence of gender differences among the limbic tracts studied suggesting that this bias was unlikely to influence the findings (Thiebaut de Schotten *et al*., 2011). Model fitting was carried out using SEM program OpenMx (Boker* et al.*, 2011, 2012), to provide heritability parameter estimates and their confidence intervals (Neale and Maes, 2011). Sources of variance in the DTI-extracted measures were divided into additive genetic (*A*) effects, shared environmental (*C*) effects, environmental influences that make the twins more similar, and non-shared or specific environmental influences (*E*) that contribute to differences between the twins and also include a possible measurement error (Plomin and Kosslyn, 2001). The proportion of variability that can be attributed to additive genetic factors is called ‘heritability’. Maximum likelihood estimates of *A*, *C* and *E* were obtained (values range from 0 to 1, where *A* + *C* + *E* = 1), with their 95% confidence intervals calculated and a series of nested models compared. A full ACE model was compared to the nested AE, CE and E models (testing the effects of common environmental factors, additive genetic factors and all familial resemblance, respectively). However, in cases where effect sizes, sample sizes or trait prevalence are low, statistical power for univariate twin analyses can become an issue (Neale *et al*., 1994) and thus only full ACE model estimates are reported. We did not specifically model familial factors, but we analysed E-only models to test for familial influences which refer to a significant combined effect of *A* + *C*. Goodness-of-fit probability for the ACE model > 0.05 suggests a good fit (the opposite of the usual *P*-value convention). Estimates are significant if the reported maximum likelihood 95% confidence intervals do not contain zero.

## Results

### Intra-class correlation coefficients

**Fig. 2.**
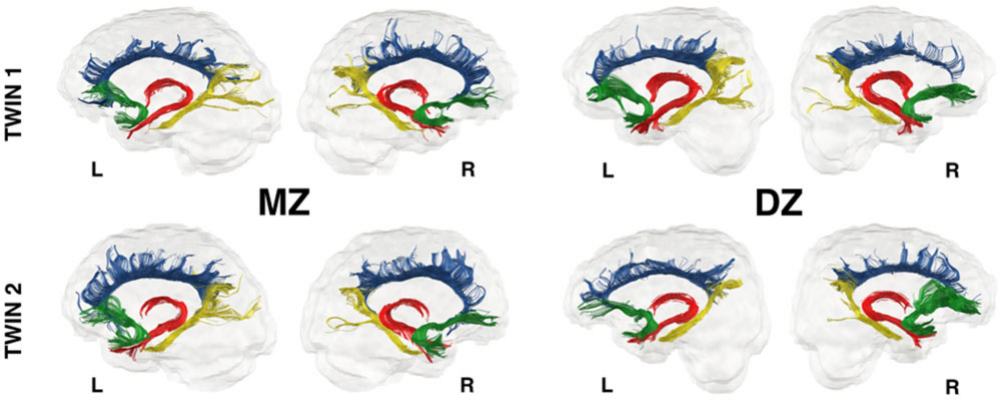
Representative examples of the four limbic tracts dissected in MZ and DZ twin pairs. The dorsal cingulum is shown in blue, the ventral cingulum in yellow, the fornix in red and the uncinate fasciculus in green colour.

The ICC results for FA, MD and volume measures extracted from four limbic tracts are shown in Tables 1–4. Overall, the ICCs for the MZ twins were either higher or similar to those of the DZ twins. In MZ twins, these correlations were statistically significant in all limbic tracts (*P* < 0.05). This may suggest high genetic or shared environmental effects on the anatomy of the limbic tracts. The ICCs of the right MD in both ventral cingulum and fornix were high and significant in both MZ and DZ twins, suggesting presence of shared environmental effects. Representative examples of the anatomy of the four limbic tracts in MZ and DZ twin pairs are shown in Figure 2. Genetic and environmental effects were formally tested using SEM.

### Structural equation modelling

**Fig. 3.**
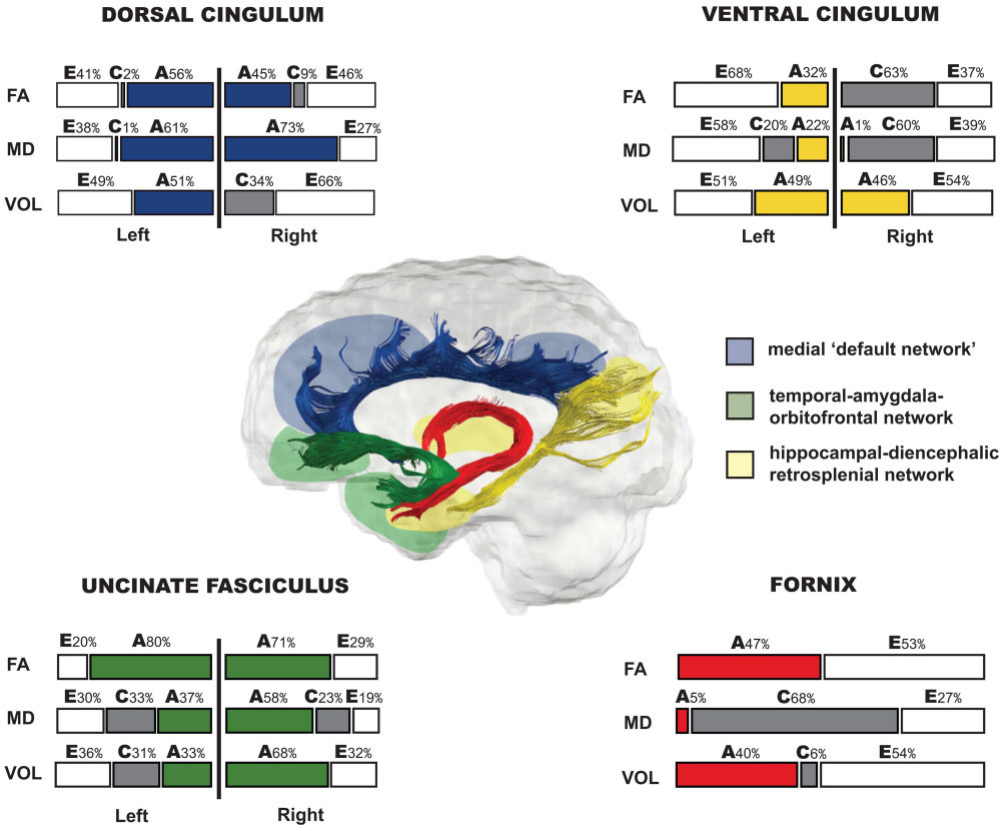
The relative contribution (in percentages) of genetic (A), shared environmental (C) and specific environmental (E) factors on the variability of the FA, MD and volume of fornix (red), the bilateral dorsal (blue) and ventral (yellow) cingulum, and the bilateral uncinate fasciculus (green).

SEM was used to extract the relative contributions of additive genetic (*A*), common environmental (*C*) and unique environmental (*E*) factors on the variability of the volume, FA and MD measures of the four limbic tracts (Tables 1–4). The width of the confidence intervals was sometimes considerable due to the relatively small sample size of our study compared to the standard behavioural genetics studies. This meant that for some measures, the individual genetic (*A*) and shared environmental (*C*) effects were non-significant, but significant when tested together as familial effect (*A* + *C*) (Wright *et al.*, 2002). The *P* values shown in Tables 1–4 indicate the fit of the ACE model compared to the saturated model; values above 0.05 indicate a good fit of the model. The results are presented according to the three-network model of the limbic system (Figure 3).

#### Heritability of dorsomedial ‘default’ network

##### Dorsal cingulum

Specific environmental factors (*E*) accounted for 49% and 66% of variance in the left and right volume of dorsal cingulum, respectively, with significant familial effect present only in the left hemisphere (51%), and smaller insignificant familial effect (36%) in the right hemisphere where the E model was the best fit. In contrast to the volume, genetics (*A*) accounted for most of the variance in the right MD (73%), while significant familial effects (*A* + *C*) accounted for 62% of left MD. Heritability of FA was high and accounted for 56% and 45% of variance in the left and right hemisphere respectively (Table 1).

**Table 1.** dCG MZ and DZ ICC coefficients of FA, MD and volume in the left and right hemisphere, with standardized additive genetic (*A*), common environmental (*C*) and unique environmental (*E*) variance components of the full ACE model (and 95% CI) with the associated DF, chi-square and *P* values

dCG	ICC (MZ)	ICC (DZ)	*A*	*C*	*E*	Best fit model*	χ^2^ (DF = 6)	*P* value
FA (L)	0.63** (0.32–0.81)	0.29 (−0.25 to 0.80)	0.56 (0–0.77)	0.02 (0–0.67)	0.42 (0.23–0.70)	(*A* + *C*)*E*	1.62	0.95
FA (R)	0.52** (0.18–0.75)	0.39 (−0.12 to 0.74)	0.45 (0–0.75)	0.09 (0–0.63)	0.46 (0.25–0.80)	(*A* + *C*)*E*	1.52	0.96
MD (L)	0.53** (0.19–0.76)	0.42* (−0.09 to 0.75)	0.61 (0–0.80)	0.01 (0–0.60)	0.38 (0.20–0.74)	(*A* + *C*)*E*	4.76	0.58
MD (R)	0.76** (0.53–0.88)	0.29 (−0.23 to 0.68)	0.73 (0.10–0.86)	0 (0–0)	0.27 (0.14–0.49)	*AE*	4.35	0.63
Vol (L)	0.60** (0.29–0.80)	0.03 (−0.46 to 0.51)	0.51 (0–0.71)	0 (0–0.65)	0.49 (0.29–0.77)	(*A* + *C*)*E*	7.81	0.25
Vol (R)	0.36* (−0.02 to 0.65)	0.35 (−0.16 to 0.72)	0 (0–0.56)	0.34 (0–0.58)	0.66 (0.42–0.96)	*E*	4.08	0.67

dCG, dorsal cingulum; L, left; R, right.

*P < 0.05, **P < 0.01, ICC test.

#### Heritability of hippocampal-diencephalic retrosplenial network

##### Ventral cingulum

Specific environmental factors (*E*) significantly explained most of the variance in FA and MD of the left ventral cingulum (68% and 58%, respectively). In contrast, the variability in FA and MD in the right hemisphere was influenced mostly by the familial factors (*A* + *C*) (61–63%), and less so by specific environment (37–39%). Variability of the volume measures was to a similar degree determined by both familial and specific environmental factors independent of the hemisphere (Table 2).

**Table 2.** vCG MZ and DZ ICC coefficients of FA, MD and volume in the left and right hemisphere, with standardized additive genetic (*A*), common environmental (*C*) and unique environmental (*E*) variance components of the full ACE model (and 95% CI) with the associated DF, chi-square and *P* values

vCG	ICC (MZ)	ICC (DZ)	*A*	*C*	*E*	Best fit model*	χ^2^ (DF = 6)	*P* value
FA (L)	0.35* (−0.03 to 0.65)	0.15 (−0.36 to 0.60)	0.32 (0–0.59)	0 (0–0)	0.68 (0.41–1)	*E*	1.62	0.95
FA (R)	0.66** (0.37–0.84)	0.59* (0.10–0.85)	0 (0–0.74)	0.63 (0–0.79)	0.37 (0.21–0.61)	(*A* + *C*)*E*	5.25	0.51
MD (L)	0.42* (0.05–0.69)	0.37 (−0.14 to 0.73)	0.22 (0–0.68)	0.20 (0–0.60)	0.58 (0.32–0.91)	(*A* + *C*)*E*	5.29	0.51
MD (R)	0.63** (0.33–0.81)	0.62** (0.20–0.85)	0.01 (0–0.74)	0.60 (0–0.77)	0.39 (0.21–0.62)	(*A* + *C*)*E*	3.41	0.76
Vol (L)	0.57** (0.24–0.78)	0.01 (−0.49 to 0.52)	0.49 (0–0.71)	0 (0–0.59)	0.51 (0.30–0.82)	(*A* + *C*)*E*	4.50	0.61
Vol (R)	0.53** (0.17–0.77)	0.05 (−0.45 to 0.53)	0.46 (0–0.70)	0 (0–0.55)	0.54 (0.31–0.88)	(*A* + *C*)*E*	4.35	0.63

vCG, ventral cingulum; L, left; R, right.

*P < 0.05, **P < 0.01, ICC test.

##### Fornix

Familial effects significantly explained most of the variability in MD measure (73%) of fornix. In contrast, familial effects had lower effect on the variability of FA and volume (47% and 40%, respectively) (Table 3).

**Table 3.** Fx MZ and DZ ICC coefficients of FA, MD and volume, with standardized additive genetic (*A*), common environmental (*C*) and unique environmental (*E*) variance components of the full ACE model (and 95% CI) with the associated DF, chi-square and *P* values

Fx	ICC (MZ)	ICC (DZ)	*A*	*C*	*E*	Best fit model*	χ^2^ (DF = 6)	*P* value
FA	0.52** (0.16–0.76)	0.06 (−0.44 to 0.54)	0.47 (0–0.72)	0 (0–0.47)	0.53 (0.27–0.90)	(*A* + *C*)*E*	3.59	0.73
MD	0.74** (0.45–0.89)	0.72** (0.36–0.89)	0.05 (0–0.78)	0.68 (0–0.85)	0.27 (0.13–0.49)	(*A* + *C*)*E*	6.50	0.37
Vol	0.48** (0.10–0.73)	0.29 (−0.21 to 0.67)	0.40 (0–0.70)	0.06 (0–0.60)	0.54 (0.30–0.89)	(*A* + *C*)*E*	3.68	0.72

Fx, fornix.

*P < 0.05, **P < 0.01, ICC test.

#### Heritability of temporal-amygdala-orbitofrontal network

##### Uncinate fasciculus

High genetic effects significantly accounted for most of the variability in the left FA (80%) and right MD (58%) of the uncinate fasciculus. It is important to note that the heritability of the right FA might be slightly inflated, because shared environmental factors might play a role as well in the 71% (ACE model was the best fit). Taken together, the familial effects explained 70–80% variability in the left, and 71–81% variability in the right hemisphere of MD and FA, respectively. Familial effects were also high for volume measures, accounting for 64% of the variance in the left, and 68% in the right hemisphere (Table 4).

**Table 4.** UF MZ and DZ ICC coefficients of FA, MD and volume in the left and right hemisphere, with standardized additive genetic (*A*), common environmental (*C*) and unique environmental (*E*) variance components of the full ACE model (and 95% CI) with the associated DF, chi-square and *P* values

UF	ICC (MZ)	ICC (DZ)	*A*	*C*	*E*	Best fit model*	χ^2^ (DF = 6)	*P* value
FA (L)	0.78** (0.57–0.89)	0.34 (−0.21 to 0.74)	0.80 (0.29–0.89)	0 (0–0.46)	0.20 (0.10–0.40)	*AE*	1.16	0.98
FA (R)	0.66** (0.38–0.83)	0.43 (−0.11 to 0.78)	0.71 (0–0.84)	0 (0–0.61)	0.29 (0.15–0.57)	(*A* + *C*)*E*	2.22	0.90
MD (L)	0.69** (0.43–0.85)	0.57* (0.07–0.84)	0.37 (0–0.83)	0.33 (0–0.76)	0.30 (0.16–0.55)	(*A* + *C*)*E*	0.66	1.00
MD (R)	0.80** (0.61–0.90)	0.58* (0.10–0.85)	0.58 (0.03–0.90)	0.23 (0–0.72)	0.19 (0.09–0.37)	*AE*	3.89	0.69
Vol (L)	0.64** (0.35–0.82)	0.54* (0.03–0.83)	0.33 (0–0.80)	0.31 (0–0.74)	0.36 (0.19–0.64)	(*A* + *C*)*E*	0.26	1.00
Vol (R)	0.63** (0.33–0.81)	0.43 (−0.11 to 0.78)	0.68 (0–0.83)	0 (0–0.62)	0.32 (0.17–0.62)	(*A* + *C*)*E*	5.80	0.45

UF, uncinate fasciculus; L, left; R, right.

*P < 0.05, **P < 0.01, ICC test.

## Discussion

In this study, we have used DTI tractography to investigate heritability of different limbic tracts important for memory and integrated socio-emotional processing. To our knowledge, this is the first twin study in healthy adults based on multiple tract-specific measures of the limbic system pathways.

We observed the highest heritability for bilateral uncinate fasciculus, where genetic and/or familial factors explained 64–80% of variance in the microstructural measures of FA, MD and volume. This tract is the main connection of the temporo-amygdala-orbitofrontal network that supports emotion processing, semantic cognition and social behaviour. Previous literature suggests an increase of the genetic effect on the FA of the uncinate fasciculus over time, with low heritability observed in neonates (Lee *et al.*, 2015; but see Geng *et al.*, 2012) and 9 year olds (Brouwer *et al.*, 2010), but moderate heritability at the age of 12 (Brouwer *et al.*, 2012). Our results suggest that genetic and familial effects might become increasingly important for uncinate fasciculus in adulthood. The findings are consistent with the recent voxel-wise report of high FA heritability in the uncinate fasciculus in adult population (Gatt *et al.*, 2012). Many neuroimaging studies suggest an increase in heritability over time for specific brain measures, such as cortical thickness (Lenroot *et al.*, 2009; van Soelen *et al.*, 2012) or white matter volume (Wallace *et al.*, 2006). The genetic effect on prosocial behaviour and empathy functions that this tract is likely to facilitate also increases with age (Ebstein *et al.*, 2010). Several mechanisms might be responsible for this observed heritability change over time. One of these includes the active genotype-environment correlation, which reflects the increasing capacity to select environments that reinforce genetic dispositions. Second, childhood environments are largely determined by parents, but declining importance of shared environment across development could lead to a reduced environmental variance and an increase in heritability estimates (Bergen *et al.*, 2007). Also, it is conceivable that measurement error decreases during this timeframe, because DTI in children is more prone to methodological and acquisition errors (motion artefacts, partial volume averaging, etc.) compared to adults. Reduced variance due to measurement error would manifest as an increase in heritability. Furthermore, the uncinate fasciculus has a prolonged maturation and its development continues well into third decade (Lebel *et al.*, 2008). An increase in heritability may reflect the new set of genes being expressed during development, or an age-dependent gene expression, as genes turn ‘on’ and ‘of’ in response to developmental cues. Many highly heritable psychiatric disorders, which are associated with the uncinate fasciculus abnormalities, have their peak age of onset during adolescence (Paus *et al.*, 2008), probably due to the age-dependent gene expression.

The high heritability of the uncinate fasciculus may have high significance for the understanding of certain neurodevelopmental conditions. We have previously reported, for example, that psychopathy is associated with reduced microstructural integrity of the uncinate fasciculus (Craig *et al.*, 2009). The finding of a strong genetic influence on this tract is consistent with studies that show psychopathic personality (Viding *et al.*, 2005; Blonigen *et al.*, 2005), childhood aggression and adult crime to be highly heritable (Moffitt, 1993), and reported difficulties in ameliorating core emotional personality deficiencies associated with this disorder by psychotherapy (Lee 1999). Future longitudinal studies of the uncinate fasciculus are needed to assess the contribution of genes and environment over time, and their interaction with maturational processes.

Our results also seem to suggest differences in the heritability of dorsal (medial default-mode network) and ventral cingulum (hippocampal-diencephalic-retrosplenial network). The variability of the microstructure (FA, MD) of the left ventral cingulum is mostly explained by specific environmental effects (58%, 68%) compared to the right ventral cingulum, whose microstructural variability is predominantly explained by familial effects (63%, 61%). Previously, differences in heritability estimates were related to differences in white matter asymmetries (Jahanshad *et al.*, 2013; Budisavljevic *et al.*, 2015), such that the tracts with higher FA exhibited higher heritability. The previously reported right lateralization of the ventral cingulum (Wakana *et al.*, 2007) could explain the observed differences in heritability. However, our study found symmetrical distribution of FA in the ventral cingulum, consistent with other reports (Malykhin *et al.*, 2008; Nakata *et al.*, 2009), thus not supporting this hypothesis. The volume of the bilateral ventral cingulum was equally affected by familial and environmental factors. Contrary to the ventral cingulum, the microstructure of the dorsal cingulum was under high genetic and familial factors independent of the hemisphere. Genetic and familial factors accounted for 73% and 62% of the variance in the right and left MD, 54% and 58% in right and left FA, and 34% and 51% in the right and left volume of the dorsal cingulum. Our FA heritability estimates for the dorsal cingulum are much higher than those noted in neonates (15–23%) (Lee *et al.*, 2015), but are in line with the heritability reported in childhood (51–54%; note however that these estimates are for the whole cingulum bundle) (Brouwer *et al.*, 2012) and voxel-wise reports in adults (Kochunov *et al.*, 2010; Gatt *et al.*, 2012; Jahanshad *et al.*, 2013). It is important to mention that FA of the cingulum has important functional relevance, and was found to be positively correlated with the full-scale and performance IQ, with common genetic factors affecting both measures (Chiang *et al.*, 2008, 2009). The only study that has also divided the cingulum into dorsal and ventral components in adult population did not investigate the same diffusion measures, and found small to moderate effects of genes on fibre orientation distribution (26–29% for dorsal and 14–33% for ventral cingulum) (Shen *et al.*, 2014). However, they also found high heritability of connections to the cingulate gyri, which most likely involve the dorsal cingulum fibres. Consistent with our results, measures of default-mode network activity that dorsal cingulum connects are also found to be moderately to highly heritable (Castellanos *et al.*, 2010; Glahn *et al.*, 2010; Fornito *et al.*, 2011). Our findings add further validity to the anatomical distinction between dorsal and ventral cingulum (Catani *et al.*, 2013a,b; Jones *et al.*, 2013), with high heritability estimates associated with the bilateral dorsal cingulum, implicated in social cognition and emotional processing (Bush *et al.*, 2000; Amodio and Frith 2006), compared to the left ventral cingulum, more related to memory functions and spatial orientation (Damasio 1989; Heimer and Van Hoesen 2006).

Together with the ventral cingulum, fornix is part of the hippocampal-diencephalic-retrosplenial network important for memory (Metzler-Baddeley *et al.*, 2011, 2012; Rolls, 2015). Fornix was under high familial effects for MD (68%), and moderate for FA and volume (47% and 46%). We did not have enough power to obtain significance for genetic and shared environmental effects individually. However, our ICC analysis suggests that genetics and shared environment might play a different role for FA and MD measures. Thus, additive genetic factors are likely to be more important for FA (MZ correlations more than double of DZ correlations), whereas shared environmental factors might affect MD more (MZ and DZ correlations equally high and significant). Previous studies on heritability of the fornix present conflicting results. DTI tractography studies show that fornix microstructure is highly heritable in neonates (43–62%) (Lee *et al.*, 2015), but not in children (18–21%) (Brouwer *et al.*, 2012). On the other hand, voxel-wise analyses in adults report both low (Jahanshad *et al.*, 2013) and high heritability (Gatt *et al.*, 2012) for the FA measure.

The implications of these findings may be significant to our understanding of heritability of social and emotional processing in terms of personality and healthy adult cognition, as well as disordered functioning. Nowadays, DTI measures are starting to be used as phenotypes for genome-wide association and linkage studies (Kochunov *et al.*, 2011; Chiang *et al.*, 2012; Jahanshad *et al.*, 2012) for identifying novel variants and molecular pathways associated with white matter microstructure. In our study, genetic influence varied among different diffusion measures, in line with the previous findings in children (Brouwer *et al.*, 2010) and adults (Budisavljevic *et al.*, 2015). Although the specific microstructural correlates are yet to be fully elucidated, diffusion measures are differentially sensitive to the degree of myelination, axonal membrane integrity, axonal density and diameter (Beaulieu, 2002; Song *et al.*, 2003; Budde *et al.*, 2007). Thus, the differences in heritability might reflect differences in physiological mechanisms underlying each measure. In the future, multivariate approaches in larger samples are needed to decipher the shared genetic and environmental effects between these variables. Finally, differences in heritability might also reflect differences in the reliability of diffusion measures, which can be influenced by acquisition sequence and parameters. For example, the FA and MD are found to be more reproducible and reliable than volume (Heiervang *et al.*, 2006; Wang* et al.*, 2012). By exploring multiple DTI measures that capture different aspects of the limbic white matter anatomy, our study may empower the search for specific genetic polymorphisms that impact the limbic white matter structure. Similarly, reliability is also important when discussing the differences in heritability among different limbic tracts. Previously, the cingulum and the uncinate fasciculus were found to exhibit more reliable diffusion measures compared to the fornix (Wang *et al.*, 2012). If this assumption is valid, the heritability estimates of the cingulum and uncinate would be inflated compared to the fornix, by reducing the variance error.

Although our study has a number of strengths it also has several limitations that should be taken into account. First, there are technical limitations of DTI tractography such as inability to solve crossing or kissing of fibres in a voxel, leading to the presence of false positives and false negatives (Dell’Acqua and Catani, 2012). However, all our limbic tracts were visually inspected to ensure that they conformed to known anatomical trajectories. It should be noted that noise in the tractography data and the ease of tracking may have increased the measurement error thus inflating the estimates of specific environment (*E*). Furthermore, standard limitations of the classical twin study design should be taken into account, such as assumptions of equal environments between MZ and DZ twins (Plomin and Kosslyn, 2001), problems with significant gene–environment correlations and interactions, lack of follow-up of the phenotypes over time, and environmental noise (Boomsma *et al.*, 2002). The assumption of the equal environment across zygosities is especially important when investigating the social brain, because the nature of socialization was shown to affect myelination in animals (Liu *et al.*, 2012) and the integrity of the uncinate fasciculus in humans (Eluvathingal *et al.*, 2006). Nevertheless, these effects were linked to severe socio-emotional deprivation, and the implications to the healthy (twin) population still remains to be established. Our study assumes similar degrees of social interaction between MZ and DZ twins. However, there is some evidence that MZ twins are treated more similarly by their parents and interact more frequently as adults than DZ twins, which could result in increased correlations for MZ *vs* DZ twins, and an overestimation of the genetic effects for phenotypes relevant to social cognition (Constantino and Todd, 2000). Nevertheless, the behavioural studies of twins reared apart argue in favour of the equal environment assumption, showing that the degree of social contact has no impact on behavioural similarity (Rijsdijk and Sham, 2002). Still, the heritability estimates from twin studies should be seen only as estimates, and should be interpreted carefully. The future studies should address the nature of social interaction in MZ *vs* DZ twins, and the effect it might have onto the limbic brain structures. Also, there is a question of whether the results of twin studies can be applicable to non-twin population, because twins are more likely than singletons to experience adverse prenatal and perinatal events that may affect brain development (Norwitz *et al.*, 2005). Studies report that there are no significant differences in the brain structure between twins and singletons in adulthood (Hulshoff Pol *et al.*, 2002; Knickmeyer *et al.*, 2011). Nevertheless, our findings are valid only for the adult population, because heritability is known to change with age (Lenroot *et al.*, 2009). For the standards of quantitative genetics, our twin study was further limited by sample size, leading to the confidence intervals of heritability estimates to be wide. Sample size impacts on model specification and this consideration dictated our choice of reporting estimates of the ACE model. A large sample is necessary to detect shared environmental (*C*) effects (Posthuma and Boomsma 2000). The reduction in power was overcome by reporting *C* and *A* (genetic) effects in combination, when they were statistically significant together, and expressing them as ‘familial effects’ (Wright *et al.*, 2002). Finally, our study would have benefited from the inclusion of neuropsychological or behavioural measurements. This would have enabled us to complete a more comprehensive investigation of behavioural–neuroanatomical heritability.

In summary, our findings indicate a strong genetic and familial effects on the anatomy of the limbic pathways that underpin emotion processing, that is, uncinate fasciculus (temporo-amygdala-orbitofrontal network) and dorsal cingulum (medial default-mode network) and a weaker but still high effect on the tracts that underpin memory, that is, left ventral cingulum and fornix (hippocampal-diencephalic retrosplenial network). In the light of the differential role that these networks have with respect to social and cognitive functioning, these findings might have important translational implications.
